# Molecular Cloning, Expression Analyses, and Physiological Roles of Cathelicidins in the Bursa of Fabricius of the Japanese Quail, *Coturnix japonica*

**DOI:** 10.3390/antibiotics12081341

**Published:** 2023-08-19

**Authors:** Takumi Ikeda, Hirotada Kondo, Daiki Nunomura, Genki Sato, Machi Ito, Nanako Yamanaka, Shawichi Iwamuro, Itaru Hasunuma, Sakae Kikuyama, Tetsuya Kobayashi

**Affiliations:** 1 Division of Life Science, Graduate School of Science and Engineering, Saitama University, 255 Shimo-okubo, Sakura-ku, Saitama 338-8570, Japan; 2 Department of Biology, Faculty of Science, Toho University, 2-2-1 Miyama, Funabashi 274-8510, Japaniwamuro@bio.sci.toho-u.ac.jp (S.I.); i.hasunuma@sci.toho-u.ac.jp (I.H.); 3 Department of Biology, Faculty of Education and Integrated Arts and Sciences, Center for Advanced Biomedical Sciences, Waseda University, 2-2 Wakamatsu-cho, Shinjuku-ku, Tokyo 162-8480, Japan; kick-yama@waseda.jp

**Keywords:** antimicrobial peptides, bursa of Fabricius, cathelicidins, quail, short-chain fatty acids

## Abstract

Antimicrobial peptides (AMPs) act directly on pathogens and maintain the anti-inflammatory effects and activation of immunocompetent cells. Therefore, the activation of the immune system in poultry via the elevation of endogenous AMPs has been attempted. In this study, we focused on the host defense mechanisms in the bursa of Fabricius (BF) of Japanese quail, cloned the cDNA of cathelicidin (CATH)-1 to -3, and analyzed their expression sites. In situ hybridization experiments revealed the mRNA expression of the CATHs in the interfollicular epithelium surrounding the lumen of the quail BF, which suggests that each CATH may exert its antimicrobial action directly in the BF. The intravenous injection of bacterial lipoteichoic acid and lipopolysaccharide endotoxins into the quail promoted the mRNA expression of CATH-1 and CATH-3 in the BF. The addition of CATH-1 or CATH-2 at the time of the antigen injection into mice resulted in antiserum with high antibody titers. Ad libitum administration of butyrate, a short-chain fatty acid, in the drinking water induced an increase in CATH-2 mRNA expression in the BF under certain conditions. These results may improve the defense mechanisms of quail by stimulating CATH expression in the BF through their diet.

## 1. Introduction

Antimicrobial peptides (AMPs) are gene-encoded polypeptides that are involved in the host defense as part of the innate immune system and are found in a wide range of organisms, including bacteria to vertebrates and plants [1]. AMPs exhibit growth-inhibitory effects, such as bactericidal and bacteriostatic effects, against pathogens. Although AMPs have no consensus sequence, most are composed of fewer than 50 amino acid residues and are positively charged in an aqueous solution as they contain many basic amino acids. AMPs also form amphiphilic secondary structures in which the hydrophilic and hydrophobic amino acids are spatially separated [2]. Because of these biochemical properties, AMPs are attracted to negatively charged substances such as lipopolysaccharide (LPS) and lipoteichoic acid (LTA) on the surface of bacterial cells, which causes cell wall lysis. In addition, AMPs exert their antimicrobial effects by acting on the lipid bilayer of the cell membrane, which causes the membrane to lose its integrity and its contents to leak out [3]. Cathelicidins (CATHs), along with defensins, are a representative family of vertebrate AMPs that are found in a variety of animal species, including humans and livestock (e.g., cattle, horses, pigs, sheep, goats, chickens, rabbits, and some species of fish) (reviewed in [4]). The CATH family is unique in that the precursor sequence is conserved between species. AMPs, including CATH, have antibacterial, antifungal, antiviral, anti-inflammatory, and wound-healing properties. Because AMPs are effective against drug-resistant bacteria, they are expected to be utilized as new prophylactic and therapeutic agents in the medical and pharmaceutical fields [5]. This includes avian AMPs [6].

Countermeasures against infectious diseases are extremely important for livestock such as poultry. Many diseases and infections are known to occur in poultry, and symptoms of these diseases range from severe to minor. In many cases, poultry infectious diseases have a significant impact on the growth and egg-laying numbers, which cause serious economic losses for producers. To prevent the onset of infectious diseases in poultry, sanitation of the rearing facilities, vaccination for prevention, and medication are used, but these measures are costly and labor intensive. Research is being conducted on the use of endogenous AMPs to protect poultry from infection [7]. In chickens (*Gallus gallus*), the presence of four AMPs belonging to the CATH family has been reported in the bursa of Fabricius (BF), and they were named fowlicidin-1, -2, and -3 and CATH-B1 [8,9]. Among them, CATH-B1 is regarded as a major AMP in the BF as it has remarkable gene expression levels in the BF, high antimicrobial activity, endotoxin-binding capacity, and mast cell-inducing activity [10]. The four CATH homologs have been cloned in other avian species such as quail, pheasants, peafowl, and ducks. The designations CATH-1, -2, and -3 are used for the homologs of fowlicidin-1, -2, and -3, respectively.

Quail is a representative poultry species that is kept in Japan in numbers that are second only to chickens for egg-laying and meat production. Regarding the CATH family in quail, the cDNA of the three CATHs (ccCATH-1–3) have been cloned from the spleen of the European quail *Coturnix coturnix* [11]. The CATH gene has also been analyzed through the *de novo* assembly of genomic DNA in Japanese quail *Coturnix japonica*, and the amino acid sequences of CATH (cjgCATH)-1–3 and CATH (cjgCATH)-B1 have been reported [12]. The antimicrobial activity and spectrum of ccCATH-1 and -2 peptides have been verified using synthetic replicates. The expression site of CATH-B1 in the BF of chickens has also been identified through immunohistochemical analysis, but similar analyses have not been performed for the other CATHs.

The BF of chicken consists of approximately 1 × 10^4^ lymph follicles composed of a cortex and medulla [13]. The epithelium surrounding the BF lumen, which is connected to the cloaca, is composed of two types of epithelial cells: namely, follicle-associated epithelium (FAE), which is capable of phagocytosis, and inter-follicular epithelium (IFE), which produces and secretes mucus [14]. External antigens that enter from the cloaca or intestinal tract are incorporated into the follicular medulla by FAE transcytosis, and the antigen is presented to undifferentiated B cells via follicular dendritic cells [15,16]. The presence of several types of CATH with antimicrobial activity in the BF implies that the BF directly protects against bacterial infection via AMPs and, furthermore, that the BF AMPs bridge the gap between the innate and acquired immune systems.

Fatty acids are composed of chain hydrocarbons and monovalent carboxylic acids, and those that have two to five carbons per chain are called short-chain fatty acids (SCFAs). SCFAs are produced in the intestinal tracts of vertebrates through the fermentation of ingested dietary fiber by the enteric bacteria present in the intestinal tract. Recently, the effects of SCFAs on the expression of AMPs such as β-defensin 9 and LL-37, a type of CATH, were analyzed using mammalian digestive organs and their strain cells, and it was reported that SCFAs enhanced the AMP mRNA expression [17]. Supplementation with SCFAs increased the quail BF weight [18], which suggests a link between SCFAs and the BF, but little is known about the involvement of SCFAs in the expression of AMPs in quails.

In this study, to elucidate the role of AMPs in the BF of Japanese quail and to develop a simple method to increase their expression, we cloned the cDNA of CATH-1, -2, and -3, measured the antimicrobial activity of CATH-1 using a synthetic peptide, and examined their expression sites through in situ hybridization and immunohistochemistry. We also analyzed the induction of CATH mRNA expression by an endotoxin and verified the enhancement of antibody production by co-injection of CATH peptides with immunogen and the promotion of endogenous CATH expression through ad libitum oral administration of SCFA.

## 2. Results

### 2.1. Cloning and Sequence Analysis of the cDNA of CATH-1, -2, and -3

**Figure 1:**
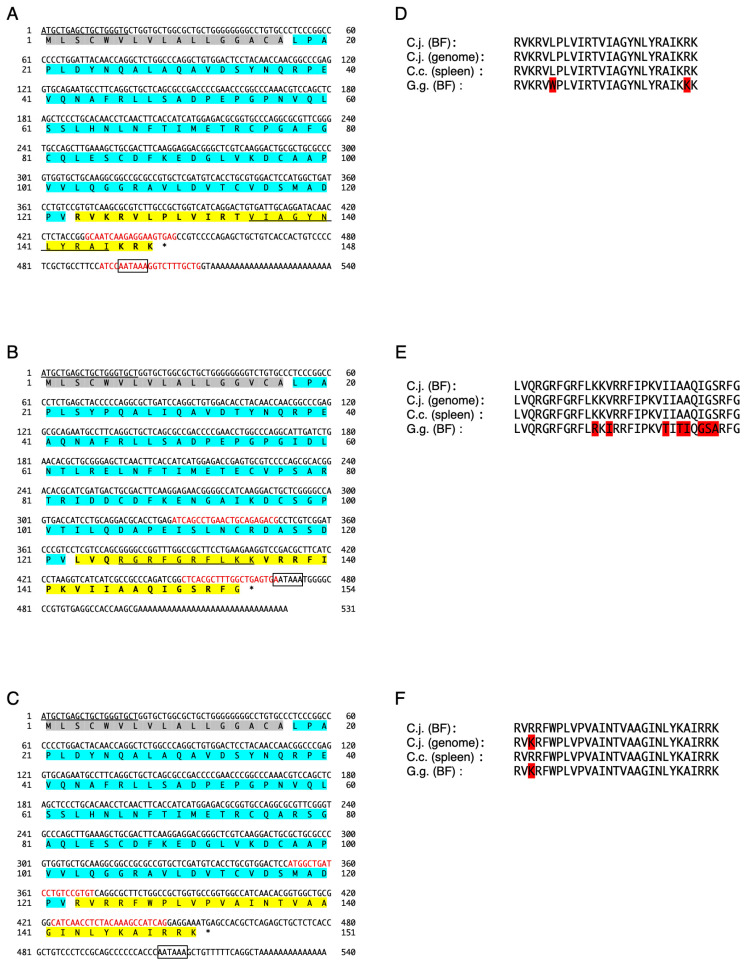
Nucleotide and deduced amino acid sequences of CATH precursor cDNA that was cloned from *Coturnix japonica* BF (**A**–**C**) and a comparison and schematic alignment of amino acid sequences of CATH-1 to -3 deduced from *C. japonica* (C.j.) BF cDNA, *C. japonica* genome DNA, *C. coturnix* (C.c.) spleen cDNA, and *G. gallus* (G.g.) BF cDNA (**D**–**F**). In the nucleotide sequences, the forward primer-derived sequences are underlined, poly-adenylation signals are in boxes, forward and reverse primers for real-time RT-PCR are in shown in red, and the stop codons are marked with asterisks (*). For the amino acid sequences (**A**–**C**), the putative signal peptide, cathelin, and mature CATH sequences are shadowed in gray, light blue, and yellow, respectively, and the sequences used for the antibody production are underlined. In (**D**–**F**), different amino acid residues are shadowed in red.

The cDNA encoding the precursor protein of CATH (cjCATH)-1, -2, and -3 was cloned from the total RNA of Japanese quail BF using the 3′-RACE method. Their nucleotide sequences and predicted sequences are shown in Figure 1A–C. The amino acid sequence of the region corresponding to CATH-1–3 was compared with those predicted from Japanese quail genomic (cjg) DNA, European quail (cc) spleen cDNA, and chicken (gg) BF cDNA (Figure 1D–F). In CATH-1, all quail sequences were identical, whereas those from Japanese quail BF and chicken BF differed by two amino acid residues out of the 26 amino acid residues. Similarly, in CATH-2, all quail-derived sequences were identical, while those from the Japanese quail BF and chicken BF differed by eight out of the 32 amino acid residues, and in CATH-3, those from the Japanese quail BF and European quail BF had identical amino acid sequences. Within Japanese quail, the genome-derived sequence and BF-derived sequence differed by one residue out of the 29 amino acid residues, and the sequence was identical to that of CATH-3 from the chicken BF. Nucleotide sequences of the CATH-1, -2, and -3 precursor cDNA clones have been deposited in the GenBank/EMBL/DDBJ database with the accession numbers LC769894, LC769895, and LC769896, respectively.

### 2.2. Antimicrobial Activity of cjCATH-1

**Figure 2:**
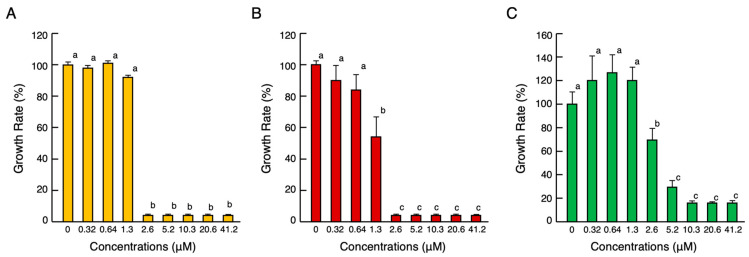
Effects of various concentrations of synthetic cjCATH-1 on the growth of Gram-negative bacteria *E. coli* (**A**), Gram-positive bacteria *S. aureus* (**B**), and fungi *C. albicans* (**C**). Cells from each bacterial or fungal strain were incubated with serially diluted cjCATH-1 for 18 h at 35 °C. Bars and vertical bars represent the means ± standard error of the mean (SEM) (*n* = 4). In all panels, the values of the lowercase letters sharing the same superscripts are not significantly different (*p* ≥ 0.05).

The cDNA cloning results showed that cjCATH-1–3 has the same amino acid sequence as ccCATH-1–3. At present, ccCATH-2 and -3 have been reported to have antimicrobial activity [11], but ccCATH-1 has not. Thus, the antimicrobial activity of cjCATH-1 (ccCATH-1) against a Gram-negative bacterium (*Escherichia coli*), Gram-positive bacterium (*Staphylococcus aureus*), and fungi (*Candida albicans*) was determined using the broth microdilution method (Figure 2A–C). The growth inhibition of all pathogen cell strains began at 1.3–2.6 µM and rapidly increased at higher concentrations. Since the minimum growth inhibition concentrations of ccCATH-2 and ccCATH-3 against the Gram-negative and -positive bacteria were both approximately 2.5 µM [11], cjCATH-1 was considered to have similar antimicrobial activity to these peptides, although they differed slightly depending on the strain.

### 2.3. Localization of cjCATH mRNAs and Peptides in the BF

**Figure 3:**
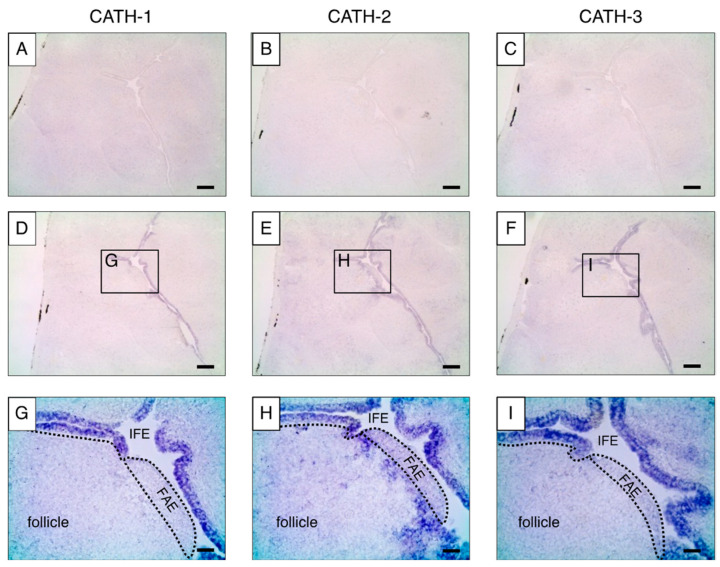
In situ hybridization of CATH-1, -2, and -3 precursors with sense (**A**–**C**) and antisense (**D**–**I**) mRNA probes in the Japanese quail BF. (**A**,**D**,**G**) CATH-1, (**B**,**E**,**H**) CATH-2, and (**C**,**F**,**I**) CATH-3. Boxes in (**D**–**F**) indicate the areas that are shown as enlarged views in (**G**–**I**), respectively. The hybridization signals of cjCATH-1, -2, and -3 were detected in the IFE, and cjCATH-2 was found in the follicles. IFE, inter-follicular epithelium; FAE, follicle-associated epithelium. Scale bars, 100 μm in (**A**–**F**); 20 μm in (**G**–**I**).

**Figure 4:**
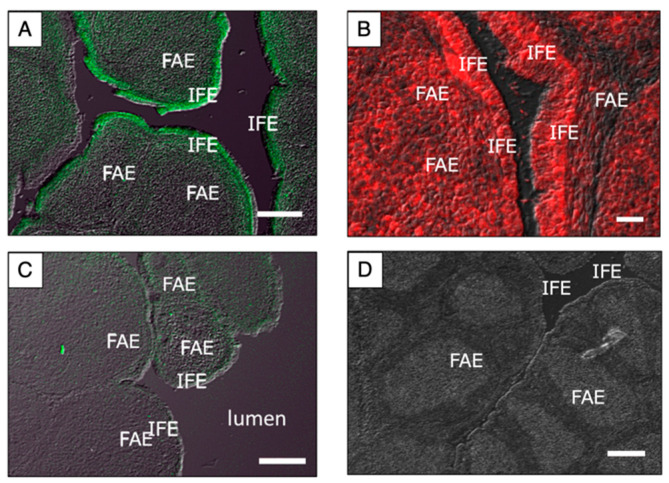
Immunohistochemical analysis of CATH-1 (**A**,**C**) and CATH-2 (**B**,**D**) in the BF of Japanese quail. Sections were pre-treated with 4% skimmed milk-phosphate buffered saline (PBS) for 1 h at 23 °C and then incubated with anti-cjCATH-1 (**A**) or anti-cjCATH-2 (**C**) antiserum overnight at 4 °C. Antigen-preabsorbed anti-cjCATH-1 (**C**) or anti-cjCATH-2 (**D**) antiserum were used as negative controls. After rinsing with PBS, the sections were incubated with FITC-conjugated donkey anti-guinea pig IgG (**A**,**C**) or AlexaFluor568 conjugated goat anti-rabbit IgG (**B**,**D**) for 2 h at 23 °C. Fluorescent signals of CATH-1 and CATH-2 were detected in the IFE, and CATH-2 was also detected in the follicles. IFE, inter-follicular epithelium; FAE, follicle-associated epithelium. Scale bars, 100 μm in (**A**,**C**), 20 μm in (**B**), and 150 μm in (**D**).

To analyze the localization of cjCATH mRNA in the quail BF, in situ hybridization using digoxigenin (DIG)-labeled RNA probes was performed on fresh frozen sections of the BF from a 3-week-old male quail (Figure 3). A comparison of the low-magnification images of the antisense and sense probes revealed specific signals only with the antisense probe. Higher-magnification images of the antisense probe showed that the cjCATH-1, -2, and -3 mRNAs were detected in the IFE, and cjCATH-2 was also found to be expressed in the follicles. On the other hand, no signals of the cjCATH-1, -2, and -3 mRNAs were observed in the FAEs. Furthermore, the localization of cjCATH-1 and cjCATH-2 in the BFs was also analyzed at the peptide level by immunohistochemistry using specific antibodies. The results showed that both cjCATH-1 and cjCATH-2 were strongly expressed in the epithelium, and cjCATH-2 was also detected in the follicles (Figure 4). Previously, Goitsuka et al. [9] showed a result of the comparison of CATH mRNA expression levels using RT-PCR, demonstrating that CATH-B1 mRNA expression was exceptionally high, which was followed by CATH-2 mRNA expression. In contrast, CATH-1 expression was weak, and CATH-3 was scarcely detected. The fact that only CATH-2 was detected in the follicles of BFs in the present study is in accord with the difference in CATH-1 and CATH-2 expression reported in the chicken BFs. These signals were attenuated or eliminated by prior treatment with the respective antiserum to the antigenic peptides, thus confirming the specificity of the antibodies. Observations regarding the site of expression of the peptides were in agreement with those of in situ hybridization.

### 2.4. Endotoxin-Inducible cjCATH mRNA Expression in the BF and Suppression by Dexamethazone (DEX)

**Figure 5:**
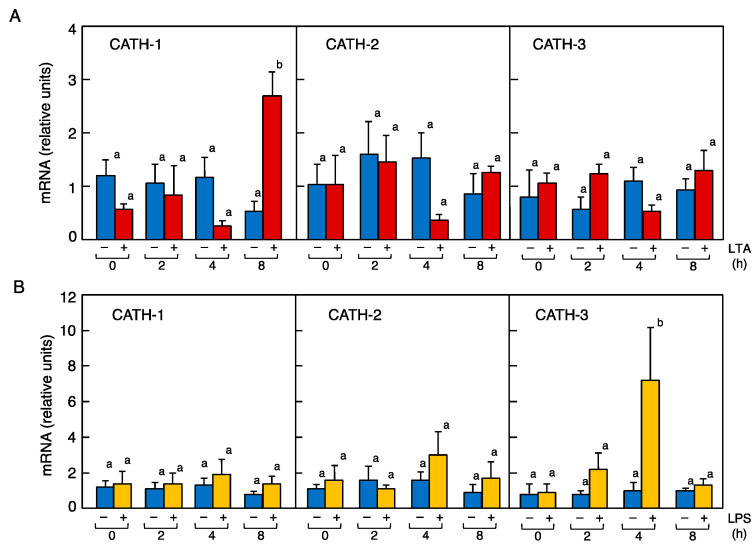
Time course effects of lipoteichoic acid (LTA) (**A**) and lipopolysaccharide (LPS) (**B**) on the steady-state levels of CATH-1, -2, and -3 mRNA in the quail BF. Male quails (*n* = 3–12) were injected with 1 mg/kg body weight of LTA or LPS through the brachial vein. PBS was used as the negative control. After 2, 4, and 8 h, BF specimens were individually collected, and the total RNA was extracted from each sample. The amounts of CATH-1, -2, and -3 mRNA were analyzed by quantitative real-time PCR, and the values were normalized to β-actin mRNA. Each column represents the average of the experiments performed in duplicate. In all panels, values of lowercase letters with the same superscripts are not significantly different (*p* ≥ 0.05).

**Figure 6:**
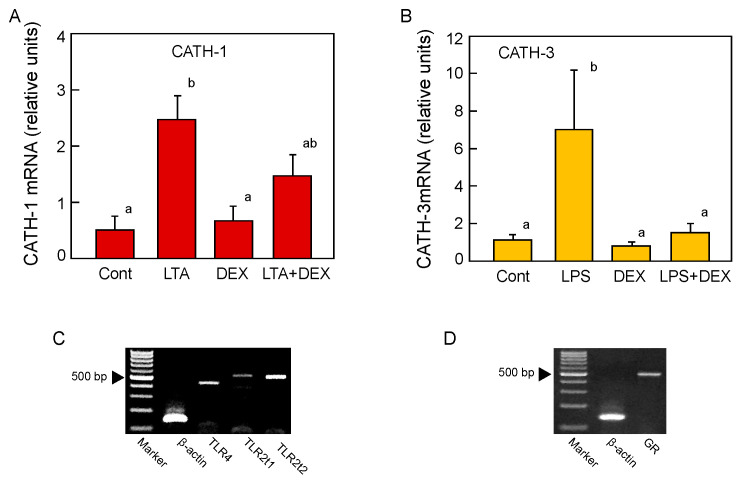
Effect of DEX on the LTA- and LPS-inducible CATH-1 (**A**) and CATH-3 (**B**) mRNA expression levels and the expression of TLR (**C**) and GR (**D**) mRNA in the quail BF. Male quails (*n* = 6–13) were injected with LTA (1 mg/kg body weight; BW) alone, LPS (1 mg/kg BW) alone, DEX (2 mg/kg BW) alone, or a combination of LTA or LPS and DEX. After 4 h, the total RNA was extracted from the BF, and the amounts of CATH-1 and CATH-3 mRNA were analyzed. Specimens of total RNA prepared from control animals were also subjected to RT-PCR using specific primers for TLR4, TLR2t1, and TRL2t2 (**C**) or GR (**D**), and the reaction products were separated by agarose gel electrophoresis. In panel (**A**,**B**), values of lowercase letters with the same superscripts are not significantly different (*p* ≥ 0.05).

LTA, a Gram-positive bacteria endotoxin, and LPS, a Gram-negative bacteria endotoxin, were administered intravenously to quail; the BF were collected 2, 4, and 8 h later; and the expression levels of cjCATH-1, -2, and -3 mRNAs were quantified by real-time RT-PCR. The LTA treatment resulted in a statistically significant increase in cjCATH-1 by about twofold at 8 h after administration, but no significant increase in the mRNA expression was detected for either cjCATH-2 or -3 at any treatment time points (Figure 5A). In the case of the LPS treatment, no significant effect on the mRNA expression was detected in cjCATH-1 and cjCATH-2, but a significant sevenfold increase was detected in cjCATH-3 compared with the untreated group at 4 h post-treatment (Figure 5B). Next, the effects of a synthetic glucocorticoid, DEX, on the LTA-induced enhanced cjCATH-1 expression and LPS-induced enhanced cjCATH-3 expression were investigated. Glucocorticoids, known as anti-inflammatory hormones, bind to their receptor, GR, in the cytoplasm and translocate into the nucleus. Ligand-bound GR binds to transcription elements in the target DNA and induces the expression of IκB, an NF-κB inhibitory protein, which suppresses the transcriptional activity of NF-κB (Ray et al. 1994) [9]. DEX suppressed the cjCATH-1 mRNA expression to a level between that of the control and LTA-treated groups, and DEX suppressed the expression of cjCATH-3 to the level of the control group (Figure 6A,B). The treatment with DEX alone did not show any significant effect on the changes in the expression of cjCATH-1 and cjCATH-3. These results suggested that there were differences in CATH responding to the different types of endotoxins in the quail BF. Since the expression level of cjCATH mRNA in the BFs was altered by the administration of LTA and LPS, it was likely that there were appropriate receptors for the endotoxins in the cells of the BFs. Accordingly, we examined the mRNA expression of TLR4 (LPS receptor), TLR2t1 (type 1 LTA receptor), and TLR2t2 (type 2 LTA receptor) in quail BF by RT-PCR. The results showed that each TLR mRNA was expressed (Figure 6C). Similarly, since binding to GR was required for DEX to exert its activity, we also confirmed the GR mRNA expression in the quail BFs (Figure 6D).

### 2.5. Influence of cjCATHs on Antibody Production

**Figure 7:**
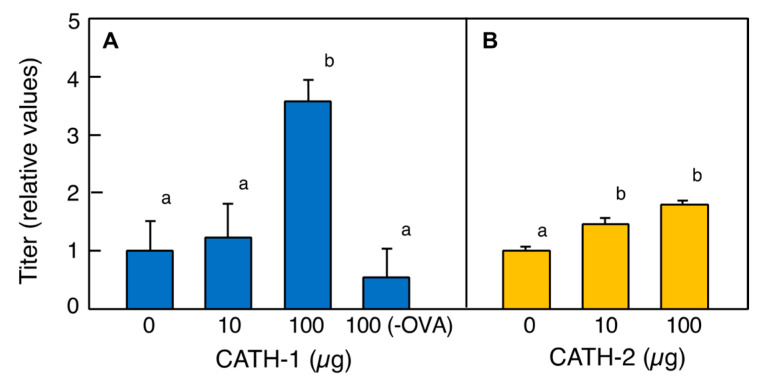
Promotive effects on the antibody production in mice through the co-administration of cjCATH-1 and cjCATH-2 with an antigen. Female mice (*n* = 3) were given an intraperitoneal injection of 500 µg of OVA in 500 µL of Hank’s Balanced Salt Solutions with 10 or 100 µg of cjCATH-1 or cjCATH-2, respectively. After 14 days, another OVA injection was administrated. After more than six days later, blood was collected from the tail vein of the mice, and the titers of the anti-OVA antibodies in the serum were measured. These titers were calculated from the relative levels to those of the control (0 µg OVA on the day 20). Values of lowercase letters with the same superscripts are not significantly different (*p* ≥ 0.05).

The presence of CATHs in the avian BF suggests that CATHs may have a role in bridging the innate and acquired immunity systems. Thus, we examined the effect of cjCATH on the antibody production in mice. When ovalbumin (OVA) was administered as an antigen, cjCATH-1 or cjCATH-2 was added to the injection solution, and the anti-OVA antibody titers in the serum collected 20 days later were measured (Figure 7A,B). The 50% value of the maximum absorbance at 450 nm (OD_450_) of the OVA-only group was used as the standard (CATH-1, OD_450_ = 0.651; CATH-2, OD_450_ = 0.642), and the dilution factor of each sample corresponding to this standard value was calculated as the antibody titer and statistically processed. The results showed that the titer of the anti-OVA antibody increased by approximately 3.5-fold in the cjCATH-1 group when 100 µg was co-administered, compared with the OVA-only group. On the other hand, when cjCATH-2 was given simultaneously with OVA, there was a slight but significant increase in the antibody titer in the 10 and 100 µg groups, but no significant dose-dependent effect was observed between these two groups (Figure 7B).

### 2.6. Promotion of CATH mRNA Expression by SCFAs

**Figure 8:**
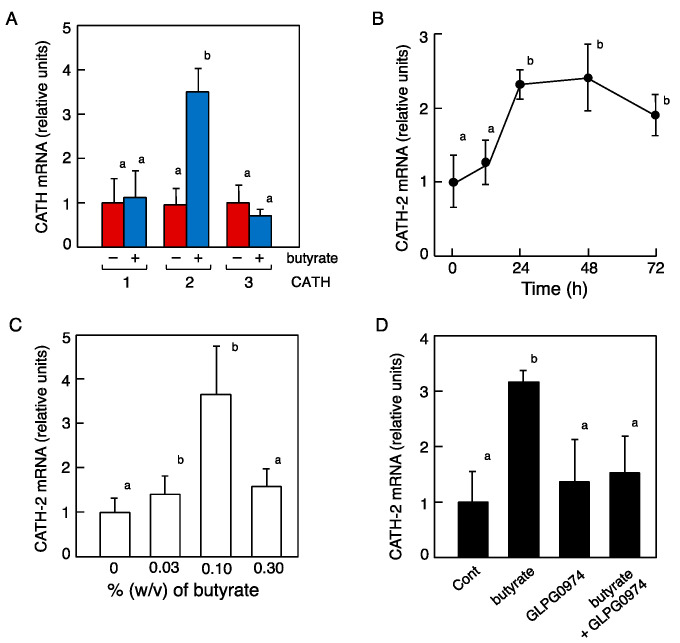
Effect of ad libitum consumption of short-chain fatty acids (SCFAs) on each of the CATH subtype mRNA expression levels (**A**), effects of treatment period (**B**), administrated dosage for 48 h (**C**), and butyrate receptor antagonist GLPG0974 (**D**) on the levels of CATH-2 mRNA expression in the quail BF time-course effects, dose-dependent effects, and in the quail BF. Male quails (*n* = 6–12) were provided with 0.1% butyrate (**A**,**B**,**D**), a type of SCFA, as drinking water ad libitum. After treatment, the BF total RNA specimens were subjected to real-time RT-PCR to measure the levels of the CATHs. The expression levels were calculated from the relative levels to those of the control (0% butyrate). In all panels, values of lowercase letters with the same superscripts are not significantly different (*p* ≥ 0.05).

The BF specimens were collected from Japanese quails fed ad libitum with butyrate solution, a type of SCFA, as drinking water (0.1%) for 48 h, and then the cjCATH-1, -2, and -3 mRNA expression levels were measured by real-time PCR. The results showed that cjCATH-2 mRNA was significantly increased compared with the SCFA non-treated group (Figure 8A). Therefore, we compared the cjCATH-2 mRNA expression levels between the different treatment times and concentrations of butyrate. The cjCATH-2 mRNA expression levels increased significantly at 24 h post-treatment with 0.1% butyrate, which did not increase or decrease significantly thereafter (Figure 8B). The treatment time was set at 48 h, and different concentrations of butyrate were administered, which indicated that 0.1% butyrate resulted in a significant increase in cjCATH-2 mRNA expression levels, whereas this increase was not observed by 0.3% butyrate (Figure 8C). The expression status of FFAR2, the SCFA receptor, in the BFs was confirmed by RT-PCR using primers designed based on the vertebrate information, and a band was observed at the target size. Therefore, the simultaneous administration of an antagonist to FFAR2 (GLPG097) suppressed the SCFA-induced cjCATH-2 mRNA expression to the level of the control group (Figure 8D).

## 3. Discussion

Since the discovery of the first antibiotic, penicillin, by Alexander Fleming in 1928, various antibiotics have been used for the treatment of infectious diseases. However, the use and misuse of large doses of antibiotics have led to an increase in drug-resistant bacteria, and there is a constant need to develop therapeutic alternatives to antibiotics, for which AMPs have traditionally been considered as prime candidates [19]. Indeed, many AMPs can directly attack pathogenic bacteria and fungi, causing cell destruction and aggregation [20]. However, the nature of the peptide substance makes the oral or injectable administration thereof impractical, and even the cream formulation of magainin, a typical AMP, has yet to reach the market (https://clinicaltrials.gov/ct2/show/study/NCT01590758, accessed on 19 July 2023). On the other hand, focusing on the indirect antibacterial effects of AMPs, many of them bind to LPS and LTA and neutralize their toxicity [20]. Furthermore, utilizing the LPS-binding ability of AMPs, their effects in suppressing LPS-dependent inflammatory cytokines have been reported [21,22,23,24]. Experiments have been attempted to quantitatively and endogenously enhance AMPs to protect against infection and maintain health, especially in livestock. Male Japanese quail are primarily raised for meat and reproductive purposes. This study focused on male Japanese quail because bacterial infection in males poses a risk not only to the meat itself but also to the spread of infection from one male to multiple females, because keeping a single male with several females is the way for reproduction.

Although Japanese and European quails are independent species, the amino acid sequences of their CATH-1, -2, and -3 are identical. Even at the precursor protein level, only two and one amino acid substitutions in cjCATH-1 and in cjCATH-2 were found in the ccCATH-1 and in ccCATH-2 cathelin regions, respectively (Figure 1). In Japanese quail, the predicted amino acid sequence of the CATH precursor protein showed a few amino acid substitutions between the genomic DNA and cDNA. This is possible because SNPs are dedicated in the genomic DNA of the Japanese quail [12]. In this study, the presence of antimicrobial activity of cj(cc)CATH-1 was demonstrated, and this indicated that all quail CATH-1–3 are antimicrobials. Japanese quail actively take in external environmental antigens through reverse peristalsis of the cloaca which sends antigens to the BF as the primary lymphoid tissue, which promotes the strengthening of the acquired immune system through immune memory [25]. The results of the in situ hybridization and immunohistochemistry experiments showed that cjCATH-1–3 mRNAs are expressed in the IFE with secretory capacity such as mucus. The IFE consists of columnar epithelial cells with many vesicles on the lumenal side of the BF, which secretes mucus containing glycoproteins and other substances into the BF lumen. Therefore, it can be inferred that CATH mRNA expressed in the IFE of the BF is translated, stored in vesicles, and released into the lumen of the BF to eliminate pathogens and other substances that have invaded from the total excretory cavity.

The detection of antimicrobial activity and the expression site suggest that CATHs in quail BF are responsive to bacteria; therefore, we administered LTA and LPS intravenously to quail to evaluate the responsiveness of each CATH mRNA expression in the BF. It is known that the direct administration of LPS into the blood of birds induces a variety of responses, including increased body temperature and the release of inflammation-induced cytokines [26]. Furthermore, mRNA expression of gallinacin-1, -7, and -12, a defensin family expressed in chick ovarian follicles, is increased by the intravenous administration of LPS [27]. Although there are few reports on the effects of LTA administration on the AMP mRNA expression in avians, in vitro experiments have shown that LPS and LTA enhanced the CATH-B1 mRNA expression in chicken BF-derived DT40 cells [28]. In the present study, CATH-1 and CATH-3 responded to LTA and LPS, respectively, and their mRNA expression levels were significantly increased in quail BFs. RT-PCR using specific primers confirmed the expression of TLR2t1 and TLR2t2, receptors for LTA, and TLR4, a receptor for LPS, in quail BFs. The TLRs are major pattern recognition receptors which play an important role in immune homeostasis against infection. Ten TLRs (TLR1LB, TLR2t1, TLR2t2, TLR3, TLR4, TLR5, TLT7, TLR15, TLR16, and TLR21) have been identified in birds, and each was localized on the plasma membrane and in endosomes [29]. Among the signaling pathways of each TLR, all TLRs except TLR3 caused the activation of nuclear factor κB (NF-κB) and mitogen-activated protein kinase, leading to a cascade reaction between the two signaling pathways. Mammalian CATH and avian gallinacin are also regulated by the TLRs-NF-κB pathway through the AMP gene transcription [29,30]. To confirm whether endotoxin-dependent CATH mRNA expression was mediated by the TLR-NF-κB pathway, we performed experiments in which DEX was added to the endotoxin, and both LTA and LPS suppressed their CATH mRNA-promoting effect. Furthermore, RT-PCR using GR-specific primers confirmed the GR expression in the Japanese quail BF, suggesting that the endotoxins-inducible CATH mRNA expression in the quail BF may be regulated by NF-κB [31,32].

When mice were intraperitoneally injected with OVA as an antigen, the co-administration of cjCATH-1 or cjCATH-2 significantly increased the titer of anti-OVA antibodies in the mouse serum. In mice, cathelin-related antimicrobial peptide (mCRAMP), a murine CATH, is expressed in the B cells and regulates the production of IgG1 [33], and LL-37, a human CATH, enhances the recognition of microbial DNA by the B cells [34]. Accordingly, it is almost certain that AMPs are involved in antibody production in local immunity. In addition, chicken CATH-2 enhances the antigen-presenting capacity in chicken monocytes and macrophages [35]. Experiments currently are being conducted to verify the increase in the antigen-presenting capacity of mouse macrophages by cjCATH and to verify the effect of cjCATH on enhancing the antibody production in the quail.

Butyrate is produced when soluble dietary fiber is metabolized by enteric bacteria in the intestinal tracts of living organisms. In chickens, butyrate has been revealed to be a strong inducer of AMP expression both in vitro and in vivo [36]. The administration of the butyrate-producing intestinal bacterium *Clostridium butyricum* increased the expression of CATH-3 and β-defensin 1 in the chicken caecum [37]. SCFA-induced AMPs in the digestive tract were suppressed in mice in which the receptor for SCFA (FFAR2) was knocked out [38]. Since FFAR2 mRNA expression was found in the BF located near the end of the intestinal tract, it is likely that the regulation of AMP expression by butyrate occurs in the same manner as in the intestinal tract [36]. Ad libitum consumption of butyrate at the appropriate concentration resulted in a significant increase in CATH-2 mRNA expression levels at 24 h in the BF and remained so until 72 h. Although it remains to be determined whether butyrate supplementation can maintain the expression of the CATH gene in quail BF permanently and whether this can increase the defense against infection, the present results indicate the way of the potential use of dietary compounds such as butyrate for the induction of AMP synthesis and the promotion of antibody production, host immunity, and disease resistance.

## 4. Materials and Methods

### 4.1. Experimental Animals and Total RNA Extraction

Male Japanese quails (*Coturnix japonica*) (3–4 weeks old) were purchased from Motoki Corporation (Tokorozawa, Saitama, Japan). All experiments were conducted in accordance with the regulations of the animal experiment committee of Saitama University after approval (H25-1-11, H26-1-22) was obtained. Each of the purchased quails was housed in a single-bird cage and provided with standard drinking water and quail feed (Feed One, Yokohama, Japan) for 24 h before it underwent the treatments necessary for each experiment as described below. The quails were sacrificed by decapitation, the BF was immediately removed, and the total RNA was extracted using a modified acid phenol/guanidine isothiocyanate procedure [39]. Recombinant DNase I (Takara, Ohtsu, Japan) was added to the total RNA solutions and incubated for 1 h at 37 °C. The absorbance was measured at 260 nm (A_260_), and the A_260_/A_280_ ratio was monitored using a spectrophotometer to determine the concentration and purity of the total RNA solutions. The sample concentrations were adjusted to 1.0 µg/µL and stored at −80 °C until use.

### 4.2. Amplification of the cjCATH Precursor cDNA by 3′-RACE

The 3′-end of the cjCATH precursor cDNA was amplified from 1 µg of the BF total RNA samples using a 3′-Full RACE Core Set (Takara) according to the manufacturer’s protocol. Briefly, the 3′-RACE reactions were performed in a 20 µL reaction solution with the Oligo dT-3 sites Adapter Primer Mix and AMV Reverse Transcriptase XL in a protocol of 30 °C for 10 min and 50 °C for 30 min for reverse transcription and then 95 °C for 15 min for the denaturation of the reverse transcriptase. For the cloning of cjCATH-1 to -3 precursor cDNAs, 10 µL of the 3′-RACE reaction products were incubated with a forward primer (5′-ATGCTGAGCTGCTGGGTGCT-3′) that was designed based on a conserved region of the signal peptides of European quail CATH-1 to -3 precursors [11] and a 3′-site adaptor primer (5′-CTGATCTAGAGGTACCGGATCC-3′) in the Core Set, a dNTP mixture, and *Ex Taq* DNA polymerase (Takara) in a 50 µL reaction solution. Subsequently, PCR was performed under the following conditions: 2 min at 94 °C for denaturation followed by 30 cycles of 30 s at 94 °C, 30 s at 62.2 °C, and 1 min at 72 °C, followed by 2 min at 72 °C for the complete extension of the DNA. The oligonucleotides for the PCR primers were provided by Eurofins Genomics Company (Tokyo). The PCR products were separated by electrophoresis on a 1.5% agarose gel, stained with ethidium bromide, and visualized using an UV transilluminator. The amplified DNA bands of appropriate sizes were excised and purified from the gel using a Freeze’N Squeeze DNA Gel Extraction Spin Column (Bio-Rad, Hercules, CA, USA), after which they were subcloned into a pSTBlue-1 vector with using the AccepTorTM Vector Kit (Novagen, Darmstadt, Germany).

Nucleotide sequence analyses were performed using the dideoxy chain termination method with the Big-Dye Terminator Cycle Sequencing Kit (Applied Biosystems, Foster City, CA, USA) by the Eurofins Genomics Company. The Basic Local Alignment Search Tool (http://blast.ncbi.nlm.nih.gov/Blast.cgi, accessed on 13 May 2023) and ExPASy (http://web.expasy.org/translate/, accessed on 13 May 2023) were used to search for homology and to perform the nucleotide-to-amino-acid sequence conversion of the sequence analysis results. In addition, multiple sequence alignment was performed using Genetyx-Mac version 15.0.1 software (Software Development Corporation, Osaka, Japan).

### 4.3. Antimicrobial Assay

A peptide with the cjCATH-1 amino acid sequence (RVKRVLPLVIRTVIAGYNLYRAIKRK) was predicted from the results of the nucleotide sequence analysis and was subsequently synthesized (Bio-Synthesis, Lewisville, TX, USA). The peptide was purified to a >90% purity and confirmed to have the correct molecular weight through mass spectrometry (theoretical value: 3715.58, measured value: 3716.3) before it was delivered in a lyophilized form. For the antimicrobial assays, *E. coli* (JCM5491), *S. aureus* (JCM2874), and *C. albicans* (JCM2085) were purchased from RIKEN BioResource Research Center (Tsukuba, Japan). The experiments were conducted in compliance with the regulations for the safety control of pathogens at Toho University and performed by authorized investigators. The antimicrobial activities of the serially diluted cjCATH-1 peptide against the animal pathogenic organisms were determined in 100 μL of cation-adjusted Mueller–Hinton broth (Becton and Dickinson, Franklin Lakes, NJ, USA). The log phase cultures (10 μL of 5 × 10^5^ colony forming units/mL) of the cells of *E. coli, S. aureus*, and *C. albicans* in 1% bovine serum albumin (BSA)-coated 96-well microtiter cell culture plates were inoculated in the supplier-recommended growth medium for secondary culture at 35 °C in normal air. After incubation for 18 h, the A_595_ was measured for each well using an iMark microtiter plate reader (Bio-Rad). Additional details are described in our previous study [40].

### 4.4. In Situ Hybridization Analysis

Aliquots of the 3′-RACE products of cjCATH-1 to -3 precursors (Section 4.2) were subcloned into a pSTBlue-1 plasmid vector, and PCR was performed to obtain the cDNA templates for the in vitro transcription. One microliter of the plasmid samples was incubated with the of cjCATH-1 to -3 cDNA-specific forward primers (Table S1) and M13 reverse primer (5′-CAGGAAACAGCTATGAC-3′) or the gene-specific reverse primer and M13 forward primer (5′-GTAAAACGACGGCCAGT-3′) and PrimeSTAR HS DNA Polymerase (Takara). The reaction conditions were as follows: 30 s at 98 °C followed by 30 cycles of 10 s at 98 °C, 10 s at 50 °C, and 5 sec for 72 °C, followed by 30 min at 72 °C. These cDNAs were used as the antisense and sense probe templates. The DIG-labeled antisense and sense probes were synthesized from the cDNAs using a DIG RNA labeling kit (Roche, Basel, Switzerland).

For the in situ hybridization analysis, male quails were sacrificed, and the BF was removed. After freeze-embedding the BF samples using Tissue-Tek O.C.T. Compound (Sakura Finetek, Tokyo, Japan), 10 µm thick sections were prepared using a Leica CM1850 cryostat (Leica, Wetzlar, Germany) and stored at −80 °C. The sections were dried thoroughly with cold air, immersed in 4% paraformaldehyde for 20 min, rinsed in PBS, and immersed sequentially in pre-acetylation solution (100 mM triethanolamine, 20 mM HCl, 0.9% NaCl; for 1 min), acetylation solution (pre-acetylation solution supplemented with 250 mM acetic anhydride; for 4 min), and 2× saline sodium citrate (SSC; 1× SSC = 150 mM NaCl and 15 mM sodium citrate, pH 7.0; for 10 min) at 23 °C. After transferring the slides to a moist chamber, 100 µL of the pre-hybridization buffer (50% formamide, 2× SSC, 1× Denhardt’s solution, 500 μg/mL yeast tRNA, 500 μg/mL heparin sodium, 0.1% sodium pyrophosphate) was placed on the section and incubated at 55 °C for 60 min. After pre-hybridization, the buffer was substituted with the cRNA probe, subjected to heat shock at 90 °C for 5 min, and diluted 100-fold with the hybridization buffer (pre-hybridization buffer supplemented with 10% dextran sulfate), and the hybridization treatment was performed overnight at 55 °C. Following the hybridization treatment, the sections were immersed in 2× SSC for 20 min at 55 °C as post-hybridization and washed with 2× SSC. The slides were then sequentially washed with 2× SSC-50% formamide at 55 °C for 1 h and at 23 °C for 20 min, 1× SSC-50% formamide at 55 °C for 1 h and at 23 °C for 20 min, 1× SSC for 20 min at 23 °C, and the pre-hybridization buffer for 10 min at 23 °C. After transferring the slides to a moist chamber, 500 µL of the blocking solution (2% Blocking Reagent, Roche; diluted in Buffer I; 100 mM Tris-HCl, 150 mM NaCl, pH 7.4) was placed on the slides for 30 min at 23 °C. The blocking solution was then substituted with 500 µL of an alkaline phosphatase-labeled anti-DIG solution (Roche) diluted in the blocking solution (1:100) and incubated at 4 °C overnight. The slides were washed with Buffer I followed by immersion in Buffer II (100 mM Tris-HCl, 100 mM NaCl, 50 mM MgCl_2_, pH 9.5) at 23 °C. The bound antibody conjugates were visualized with nitroblue tetrazolium chloride (Sigma-Aldrich, St. Louis, MO, USA) and 5-bromo-4-chloro-3’-indolylphosphatase p-toluidine salt (Sigma-Aldrich) in Buffer II at 23 °C for 2 h in the dark. After sufficient color development, the sections were washed with a reaction-terminating solution (100 mM Tris-HCl, 50 mM EDTA, pH 8.0) and embedded with VectaMount AQ Aqueous Mounting Medium (Vector Laboratories, Burlingame, CA, USA). The sections were viewed using a Leica DM750 microscope (Leica).

### 4.5. Immunohistochemistry

Peptides with the corresponding amino acid sequences to ^13–33^cjCATH-1 (VIAGYNLYRAI) and ^4–13^cjCATH-2 (RGRFGRFLKK) were introduced as an additional cysteine residue to the C-terminus, synthesized, and used for the production of anti-cjCATH-1 and -2 antibodies. These peptides were conjugated with keyhole limpet hemocyanin (KLH), a carrier protein, for use as antigens. A guinea pig and rabbit were inoculated with 0.15 mg of KLH-conjugated cjCATH-1 and cjCATH-2 fragments for the production of anti-CATH-1 and anti-CATH-2 antibodies, respectively. The synthesis of the antigen peptides, KLH conjugation, and preparation of the antibodies as well as the affinity purification were performed by Protein Purity Co (Isezaki, Gunma, Japan). Fresh frozen sections (10 µm) of the quail BF were fixed in 4% paraformaldehyde diluted in 50 mM PB, pH 7.4, for 20 min, washed with 10 mM PB, and incubated for 30 min at 23 °C with 5% normal goat serum (Dako, Agilent, Santa Clara, CA, USA) in PBS with Tween20 (PBS-T; 10 mM PB, 0.9% NaCl, 0.05% Tween20) for blocking, followed by overnight incubation with the affinity-purified anti-CATH-1 (diluted 1:50) or anti-CATH-2 (diluted 1:20) antibodies at 4 °C. The sections were washed with 10 mM PBS-T and allowed to react with the fluorescein isothiocyanate (FITC)-conjugated donkey anti-guinea pig IgG (Bioss, Boston, MA, USA; diluted 1:500) and AlexaFluor568-conjugated goat anti-rabbit IgG (Invitrogen, Thermo Fisher, Waltham, MA, USA; diluted 1:600) to detect the anti-CATH-1- and anti-CATH-2-derived signals, respectively. After washing with 10 mM PBS, the sections were mounted with FLUOROMOUNT (Diagnostic BioSystems, Pleasanton, CA, USA) before observation using an FV-1000D confocal laser microscope (Olympus, Tokyo, Japan).

### 4.6. Quantification of CATH mRNA Expression Levels by Real-Time PCR

Male quails were maintained under light conditions overnight with ad libitum access to feed and drinking water. The quails were randomly assigned to control (PBS), LTA, and LPS groups. Solutions of *S. aureus*-derived LTA (Merck, Darmstadt, Germany) and *E. coli* 026: B6-derived LPS (Merck) at 0.5 mg/mL were injected into the brachial vein of the quails to administer a dose of 1.0 mg/kg body weight (BW). The BF samples were collected at 0, 2, 4, and 8 h after administration of the PBS, LTA, or LPS, and the total RNA was extracted. The CATH-1 to -3 mRNA expression levels were found to be significantly increased 8 h after LTA administration and 4 h after LPS administration. To examine the effects of a glucocorticoid on the mRNA expression levels under these conditions, DEX (dexamethasone sodium phosphate, Wako) was co-administered with LTA and LPS. A 1.0 mg/mL DEX solution was prepared, and 2.0 mg DEX/kg BW was administered. The quails were assigned to the control, LTA, LPS, DEX, LTA + DEX, or LPS + DEX groups. After treatment, the BF samples were collected, and the total RNA was extracted as described in Section 4.2. 

The first strand cDNAs were amplified from 1 µg of the total RNA samples; they were denatured for 10 min at 70 °C and synthesized using M-MLV Reverse Transcriptase (Thermo Fisher) with random primers (Takara) according to the manufacturer’s protocol. The reaction conditions were as follows: 10 min at 20 °C, 60 min at 37 °C, and 5 min at 94 °C. Quantitative PCR was conducted using a LightCycler 1.5 instrument and Capillaries (Roche) with a 20 µL reaction. Standards for the quantification were prepared as a fivefold dilution series, and the first strand cDNA samples were diluted 50-fold before use. For the samples, 10 µL of SYBR premix *Ex Taq* (Takara), 0.8 µL of 5 µM of the gene-specific forward and reverse primers, and 6.4 µL of Milli-Q water were combined and added to each capillary. Then, 2 µL of a standard or sample was added to each capillary to determine the mRNA expression levels analysis under the following reaction conditions: 30 s (20 °C/s) at 95 °C for the initial denaturation and then 40 cycles of 5 s at 94 °C (20 °C/s) and 20 s at 65 °C (20 °C/s), followed by 0 sec at 95 °C (20 °C/s), 15 s at 65 °C (20 °C/s), and 0 s at 95 °C (0.1 °C/s) for the melting curve analysis. The CATH-1 to -3 mRNA expression levels were corrected relative to the β-actin mRNA expression levels. Nucleotide sequence information for the primers of cjCATH-1 to -3 and β-actin is shown in Table S1. 

### 4.7. Amplification of Partial cDNAs of TLR and GR by RT-PCR

After conducting the reverse transcription reactions as described in Section 4.6, the first strand cDNA samples that were prepared from the quail BF treated with LTA, LPS, or DEX or their combinations were subjected to PCR using *Ex Taq* (Takara) with sets of gene-specific forward and reverse primers for TLR4, TLR2t1, TLR2t2, GR, and β-actin (Table S1) in a 20 µL reaction mixture. The PCR was performed under the following conditions: 2 min at 94 °C for denaturation followed by 30 cycles of 30 s at 94 °C, 30 s at 62.2 °C, and 1 min at 72 °C, followed by 2 min at 72 °C for the complete extension of the DNA. Oligonucleotides for the PCR primers were provided by Eurofins Genomics Company (Tokyo, Japan). The PCR products were separated by electrophoresis on a 1.5% agarose gel, stained with ethidium bromide, and visualized using an UV transilluminator. The nucleotide sequences of the amplified DNA were confirmed by the Eurofins Genomics Company.

### 4.8. Antibody Production-Promotion Assay 

To examine the promotive effects of quail CATHs on the antibody production in mice through the co-administration with an antigen, female mice (Slc:ddY, 8-week-old, *n* = 3) were intraperitoneally injected with 500 µg of OVA (Wako) in 500 µL of Hank’s Balanced Salt Solutions with 10 or 100 µg of cjCATH-1 or cjCATH-2, respectively. After 14 days, another OVA injection was administrated. More than six days later, blood was collected from the tail vein of the mice, and the titers of the anti-OVA antibodies in the serum were measured by an enzyme-linked immunosorbent assay. Briefly, 100 µL aliquots of OVA (1 mg/mL in 10 mM calcium carbonate buffer) were added to each well of a 96-well MaxiSorp Immunoplate (Thermo Fisher) and allowed to incubate overnight at 4 °C to allow the OVA to adhere to the plate. After blocking with 1% skimmed milk in PBS, 100 µL aliquots of the serum samples diluted in a 10-fold series were added to the wells and incubated at 4 °C overnight. After washing with PBS, the wells were allowed to react with rabbit anti-mouse IgG antibody (Dako) for 2 h at 23 °C. The excess fluid was aspirated off, and the wells were washed with PBS-T and incubated with horse radish peroxidase-conjugated goat anti-rabbit IgG antibody (Dako) for 2 h at 23 °C. The excess fluid was aspirated from the wells, washed with PBS, and then reacted with 100 μL of the chromogenic substrate, 3,3′,5,5′-tetramethylbenzidine (Sumitomo Bakelite, Tokyo, Japan), and 0.3% hydrogen peroxide for 5 min. The reaction was stopped by the addition of 100 μL of 2 M sulfuric acid. Finally, the A_450_ of the samples was measured using a microtiter plate reader.

### 4.9. Promotion of CATH mRNA Expression by SCFAs

Male quails were fed solutions of 0, 0.03, 0.10, or 0.3% butyrate (Wako), a type of SCFA, ad libitum in their drinking water and maintained under continuous illumination conditions for 0, 12, 24, or 72 h. After treatment, the BF samples were collected, and the total RNA was extracted. Similarly, the quails were fed 0.1% butyrate solution ad libitum for 48 h, and 0.5 mg/mL of the butyrate receptor FFAR2 antagonist GLPG0974 (1 mg/kg BW) was orally administered at 0, 12, 24, 36, and 48 h after initiation of the butyrate solution feeding. Six hours after the last administration, the BFs were collected, and the total RNA was extracted and subjected to real-time PCR to measure the CATH mRNA levels.

### 4.10. Statistical Analysis 

The statistical analysis for the antimicrobial assay was performed using an analysis of variance followed by multiple comparisons using Scheffé’s *F*-test. For the other experiments, the statistical comparisons between different experimental groups were performed using the *F*-test followed by the Tukey–Kramer multiple comparison test, and the significance of the differences between the groups was analyzed. A value of *p* < 0.05 was considered to be statistically significant.

## 5. Conclusions

We cloned and sequenced the cjCATH-1 to -3 cDNA of the BF of Japanese quail. *C. japoica* and cjCATH-1 to -3 have the same amino acid sequence as that of *C. coturnix* ccCATH-1 to -3. The synthetic replicate of cjCATH-1 exhibited antibacterial activity against *E. coli*, *S. aureus*, and *C. albicans*. In situ hybridization experiments revealed CATH mRNA expression in the IFE of the lumen of the quail BF. The intravenous injection of LTA and LPS promoted the expression of CATH mRNA in the BF of quail. The addition of CATHs at the time of antigen injection into mice resulted in the production of antiserum with a high antibody titer. The ad libitum administration of butyrate in the drinking water induced an increase in the CATH mRNA expression in the BF of quails. These results may improve the immune defense mechanisms of quail by stimulating the CATH expression in the BF through supplementation to their diet.

## Data Availability

Not applicable.

## Supplementary Materials

The following supporting information can be downloaded at https://www.mdpi.com/article/10.3390/antibiotics12081341/s1: Table S1; Nucleotide sequences for primers used in this study.
