# Supplementary material for: Molecular Cloning, Expression Analyses, and Physiological Roles of Cathelicidins in the Bursa of Fabricius of the Japanese Quail, Coturnix japonica
Source: Antibiotics (Basel). 2023 Aug 19;12(8):1341. doi: 10.3390/antibiotics12081341 (PMC10451233; doi:10.3390/antibiotics12081341)
Supplement: Supplementary file 1 [file antibiotics-12-01341-s001.zip › antibiotics-2530360-supplementary.pdf]

Table S1. Primer sequences used in this study

Primer	Sequence (5′ to 3′)	Application
CATH common forward	ATGCTGAGCTGCTGGGTGCT	3′-RACE
3′-site adaptor	CTGATCTAGAGGTACCGGATCC	3′-RACE
CATH-1 (F)	GCTGTGGACTCCTACAACCAAC	cRNA probe production for ISH
CATH-1 (R)	TGATGACCAGCGGCAAGAC	cRNA probe production for ISH
CATH-2 (F)	CAGGCTGTGGACACCTACAAC	cRNA probe production for ISH
CATH-2 (R)	GCCCCATTTATTCACTCAGC	cRNA probe production for ISH
CATH-3 (F)	TGTGGACTCCTACAACCAACG	cRNA probe production for ISH
CATH-3 (R)	TGATGGCTTTGTAGAGGTTGATG	cRNA probe production for ISH
M13 (F)	GTAAAACGACGGCCAGT	cRNA probe production for ISH
M13 (R)	CAGGAAACAGCTATGAC	cRNA probe production for ISH
CATH-1 (F)	GGGCAATCAAGAGGAAGTGAG	Real-time PCR
CATH-1 (R)	CAGCAAAGACCTTTATTGGATGG	Real-time PCR
CATH-2 (F)	ATCAGCCTGAACTGCAGAGACG	Real-time PCR
CATH-2 (R)	TCACTCAGCCAAAGCGTGAG	Real-time PCR
CATH-3 (F)	ATGGCTGATCCTGTCCGTGT	Real-time PCR
CATH-3 (R)	CTGATGGCTTTGTAGAGGTTGATG	Real-time PCR
β-actin (F)	GAACCCCAAAGCCAACAACAGAG	Real-time PCR
β-actin (R)	CCATCACCAGAGTCCATCACA	Real-time PCR
TLR4 (F)	ACGTCACCACTATTCGGTTGG	RT-PCR
TLR4 (R)	AAGGAGGAGAAAGACAGGGTAGGT	RT-PCR
TLR2t1 (F)	ACTGGTTCAGAACTCTACTCTTCC	RT-PCR
TLR2t1 (R)	AGATTACTTATATATCTTGCAGCCTGT	RT-PCR
TLR2t2 (F)	TGAGAATTATGTCTTCCTCTTTCGC	RT-PCR
TLR2t2 (R)	GATGAAACAAGCTTTTACCTGTCATTT	RT-PCR
GR (F)	GCTCTTCTCCATCCACAACCTC	RT-PCR
GR (R)	CCACTTGCCGTCCTCCTAAC	RT-PCR
FFAR2 (F)	GCCCCATAGCAAACTTCT	RT-PCR
FFAR2 (R)	GGGCAGCCATAAAGAGAG	RT-PCR

F, forward primer; R, reverse primes.
